# Testing Adaptive Hypotheses of Convergence with Functional Landscapes: A Case Study of Bone-Cracking Hypercarnivores

**DOI:** 10.1371/journal.pone.0065305

**Published:** 2013-05-29

**Authors:** Zhijie Jack Tseng

**Affiliations:** 1 Department of Biological Sciences, University of Southern California, Los Angeles, California, United States of America; 2 Department of Vertebrate Paleontology, Natural History Museum of Los Angeles County, Los Angeles, California, United States of America; Raymond M. Alf Museum of Paleontology, United States of America

## Abstract

Morphological convergence is a well documented phenomenon in mammals, and adaptive explanations are commonly employed to infer similar functions for convergent characteristics. I present a study that adopts aspects of theoretical morphology and engineering optimization to test hypotheses about adaptive convergent evolution. Bone-cracking ecomorphologies in Carnivora were used as a case study. Previous research has shown that skull deepening and widening are major evolutionary patterns in convergent bone-cracking canids and hyaenids. A simple two-dimensional design space, with skull width-to-length and depth-to-length ratios as variables, was used to examine optimized shapes for two functional properties: mechanical advantage (MA) and strain energy (SE). Functionality of theoretical skull shapes was studied using finite element analysis (FEA) and visualized as functional landscapes. The distribution of actual skull shapes in the landscape showed a convergent trend of plesiomorphically low-MA and moderate-SE skulls evolving towards higher-MA and moderate-SE skulls; this is corroborated by FEA of 13 actual specimens. Nevertheless, regions exist in the landscape where high-MA and lower-SE shapes are not represented by existing species; their vacancy is observed even at higher taxonomic levels. Results highlight the interaction of biomechanical and non-biomechanical factors in constraining general skull dimensions to localized functional optima through evolution.

## Introduction

Convergent evolution is a prominent feature of mammalian evolution in the Cenozoic, so much so that many cases (e.g. convergently fossorial, arboreal, herbivorous, or carnivorous forms) have become textbook examples for the concept in evolutionary biology [1]. Morphological convergence is often interpreted as being adaptive for the very reason that they appeared in unrelated clades of species. This study addresses two questions about macroevolutionary morphological convergence in mammalian skull morphology: (1) Are morphologically convergent species actually convergent in functional capability? (2) If so, do those morphologies occupy local optimal peaks in a “functional” landscape? These questions are explored with theoretical morphology and finite element modeling in a case study of bone-cracking carnivorous mammals.

Adaptation, like convergent evolution, is a central concept in evolutionary biology. Studies of patterns and processes of adaptation on the macroevolutionary scale often rely on morphological characters, essentially those that are preserved in the fossil record. The concept of the fitness (or adaptive) landscape, as originally proposed to visualize possible evolutionary pathways of genetic interactions, has been adopted as a framework to examine morphology in evolutionary and ecological contexts [2]–[5]. In a demonstration of the concept at its extremes, Kauffman [6] used simulations of hypothetical genetic interactions to create two fitness landscapes, one (“Fujiyama” landscape) with a single adaptive peak, and the other with a random distribution of equally adaptive peaks. Adaptive evolution is thought to proceed on intermediate landscapes between those extremes, with differentially elevated adaptive peaks, some of which act as “topological attractors” where examples of convergence can be sought [7], [8].

In conventional morphometric studies, examples of convergent morphological evolution can be identified by macroevolutionary pathways that move toward each other in empirical morphospace, a morphospace built using existing, observed morphological diversity [8], [9]. However, convergent morphologies can also evolve via parallel evolutionary pathways that do not exhibit obvious trends of such movement in empirical morphospace [10]. The complex craniodental system of vertebrates, particularly those of mammals with heterodont dentition, is subject to multiple functional demands not only of mastication and food acquisition, but also a range of sensory functions [11], [12]. Understanding key evolutionary drivers of functional changes in such complex systems can be daunting, although there is some evidence of modularity to indicate that certain complex features evolved as integrated units [13], [14]. To put the issue at hand as an analogy in engineering optimization theory, the number of possible designs of an engineered tool is proportional to the multiplicity of functions it is intended to serve; selective pressures on multi-tasking biological structures may similarly have resulted in equally fit morphologies on distinct (but comparable) adaptive peaks in a fitness landscape [15]. This phenomenon of “many-to-one” form-function relationship has been recognized as a major feature of adaptive evolution [16].

With the aid of computer-based simulation tools, questions that surround the functional aspect of morphological evolution can now be addressed with the creation of form-function landscapes based on hypothetical morphospace [8]. The bulk of previous work on theoretical morphospace has been done in studies of plants and invertebrate animals [8], [17], [18]. Complex mathematical models have been constructed to simulate growth patterns and possible (but sometimes non-existent) morphotypes in a variety of organismal groups. However, few studies have focused on constructing hypothetical morphospaces of vertebrates, particularly mammals (but see [19]). One factor in the paucity of such studies may lie in the large number of skeletal elements that exist in vertebrates, and the highly integrated functionality of many larger animals. Such emergent properties make parameterization of key morphological traits difficult. Nonetheless, the exploration of form and function using empirical morphospace and simulation of morphotypes that occur in different regions of such morphospace has already been proposed and explored in vertebrates [19], [20]. A subset of functional simulations currently rely on finite element analysis (FEA), a technique which has gained wide use in the study of vertebrate biomechanics, particularly on the craniodental system [21], [22]. However, FEA has mostly been applied to studies of existing or fossil morphology, and has not been used with emphasis on theoretical morphology. This study aimed to explore the union of functional simulations of craniodental function using FEA with the study of theoretical morphology using functional landscapes and hybrid morphospaces. A prominent example of convergent evolution in the Cenozoic record of mammals, that of bone-cracking hyaenids and borophagine canids, was used to demonstrate the utility of combining functional and theoretical approaches to study evolutionary (and potentially adaptive) changes in morphology. As this study attempts to demonstrate, the use of such a theoretical framework to test adaptive hypotheses regarding convergent morphologies, by comparing realized forms with a range of theoretically possible ones, provides new insights into the nature of constraint and adaptive function in the evolution of the carnivoran skull.

### Finite element analysis

FEA was originally an engineering technique, used in the design process to conduct mechanical testing on simplified, discrete representations of real-world objects. The term was coined by Clough [23] for applications in the civil engineering field. In the past two decades, the application of FEA to studies of vertebrate functional morphology has seen a notable increase, particularly in the study of the craniodental system [22], [24]–[26]. Application to mammalian craniodental biomechanics has been tested in a diverse range of research questions, from convergent evolution [27], ecological niche [28], conservation biology [29], bite force [30], to bone strain and model validation [31], among others.

The initial input to FEA is the morphology of interest, either derived from computer-generated models, photos of specimens, or more commonly, computed tomography (CT) images [21]. The representations of the morphology in question are modified and converted into element meshes, which are mathematical, geometric constructs of the original morphology. Material properties and boundary conditions are assigned to the mesh model with values derived from experiments, or in the case of extinct organisms, experimental values taken from closely related living taxa [21]. FE analysis software programs can then perform simulations of forces on the FE model, returning results in the form of stresses, strains, and bite force [32]. The process of improving models of actual species, usually by digitally repairing incomplete areas of the structure of interest, is amenable to manipulation and creation of non-existing, theoretical shapes that can then be tested in the same way as a model of an actual species.

### Bone-cracking ecomorphology

Ecomorphologies are categories of ecological specialization, based on characteristic morphological features inferred to be associated with specific functional capabilities. The repetitive evolution of major ecomorphologies in carnivorous mammals is a key feature of this mammalian group throughout their Cenozoic evolution [33]–[36]. As in stereotypical cat-like and dog-like carnivorans, the hyena-like forms are hypercarnivores specialized in consumption of vertebrate flesh [34]. These hyena-like forms also have robust craniodental morphological features that are seen as adaptations for durophagy. Strong and bulbous cheek teeth, deep and often rounded foreheads, and large, rugose parietal areas for jaw muscle attachment are the main features of bone-cracking ecomorphologies [34], [37]. These morphological features are associated with impressive bone-cracking capability in the extant spotted hyenas [38], [39]. The generally large-bodied carnivorans that possess these morphological features have been identified in the fossil record in Hyaenidae [40], borophagine canids [41], and Percrocutidae [42]–[44].

Hyaenids and percrocutids are feliform carnivorans, with the majority of their evolutionary record in the Old World [40]. The earliest records of both groups are found in middle Miocene deposits of Eurasia; percrocutids did not survive the Miocene, whereas hyaenids are known today by four species, composing one of the smallest living carnivoran families [45]. Distinct morphological differences between percrocutids and hyaenids, which have been proposed to be sister groups, are established at their earliest occurrences [46]. The evolution of true hyaenids have been demonstrated to be quite gradual, with sequential appearance of six ecomorphological categories through their ∼25 m.y. fossil record [40], [47]. In contrast, the fragmentary fossil record of percrocutids is currently lacking a comprehensive phylogenetic framework. Nevertheless, it is clear that the most robust forms in either lineage, the hyaenines and the percrocutid *Dinocrocuta*, respectively, possessed full capability for bone-cracking comparable to, or exceeding, the modern spotted hyena (*Crocuta crocuta*) [43].

**Figure 1:**
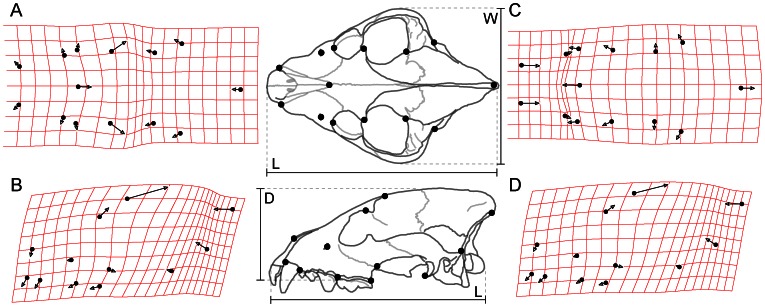
Convergent evolution of skull shapes in dogs and hyenas. Data for borophagine canids (A–B) and hyaenids (C–D) from two-dimensional geometric morphometric analyses in [10]. A, C, dorsal views; B, D, lateral views. Illustrations of skull show the measurements of width to length (W∶L) and depth to length (D∶L) taken from theoretical and actual skull shapes.

Canidae are North American natives, evolving into three subfamilies that represent some of the most common fossil carnivorans to be found in the Tertiary: Hesperocyoninae, Borophaginae, and Caninae [41], [48], [49]. All modern canids belong in Caninae, with no surviving species from the other two subfamilies [50]. Borophaginae contain the most hyena-like canids, some of which have long been considered ecological vicars of true Old World hyaenids [37], [41], [51]. Craniodental function in the most specialized borophagine canids has also been shown to resemble those of hyaenids in bone-cracking capability [52]. Furthermore, the bone-cracking ecomorphologies in the borophagine canids evolved derived craniodental morphology via parallel evolutionary pathways alongside the macroevlutionary patterns observed in hyaenids (Fig. 1, [10]). Such extensive convergence in craniodental morphology and inferred functional capability proceeded under a complex interplay of adaptation and constraint [10], [37], [53].

Taking the evolutionary patterns observed previously for borophagine canids, hyaenids, and percrocutids, I test the hypothesis that bone-cracking ecomorphologies were specialized forms that converged on identical or equivalent functional peaks on a simplified form-function landscape. Form and function are closely linked, so the functional pathways shared by bone-cracking ecomorphologies should reflect their parallel evolution in skull shape changes. Secondly, the convergently evolved specialist species in both lineages occupy optimal peaks in the theoretical morphospace containing a wide range of possible morphologies. A novel “functional” landscape built using principles of functional morphology and theoretical morphology is presented as a framework to test these hypotheses. The general utility of such approach is then demonstrated by tracking evolution of craniodental function, as inferred from FEA simulations, of actual fossil and extant species in the three carnivoran lineages discussed above.

## Materials and Methods

No permits were required for the described study, which complied with all relevant regulations. All specimens, except for the skull of *Proteles cristata*, are in recognized museum collections listed in the Supplementary Information section. The dry skull of the extant aardwolf *Proteles cristata* was purchased from a natural history company (Necromance, 7220 Melrose Ave, Los Angeles, CA 90046) which sells specimens that are “legally obtained by-products and can be legally sold according to California state laws”. The relevant California Penal Code 653o and 653p do not prohibit the import of *Proteles cristata*, which is listed by CITES Appendix III as a species of least concern in Botswana. In addition, the aardwolf is not listed under the federal Endangered Species Act foreign species list. The specimen, of unknown provenance other than “southern Africa”, is being used as a destructive sample in a separate study, and was CT-scanned for the current study prior to destruction. The raw CT dataset, which can be used to reconstruct the original morphology of the destructed specimen, is deposited online in Dryad (doi:10.5061/dryad.r2b1h). All theoretical and actual species models generated in this study are also available in Dryad at the above DOI address.

### Hybrid morphospace

Strictly speaking, a theoretical morphospace, as defined by McGhee [8], is constructed without any morphometric input from actual specimens. The geometric shapes of organismal morphology are created using mathematical models, spanning a range that may encompass non-existent shapes [8]. In contrast, the hybrid morphospaces in this analysis were constructed with an actual ecomorphology: the jackal-like *Ictitherium* [40], [47]. This fossil hyaenid provided a morphology that resembled less specialized forms of the convergent bone-cracking lineages, and therefore is a good starting point to examine how morphological evolution proceeded toward specialized forms. A two-dimensional morphospace was then used in conjunction with two functional properties (*sensu* [16]), described below, to create form-function landscapes. The morphological parameters were chosen to represent the main axes of evolutionary skull shape changes observed in both the Hyaenidae and the borophagine canids (Fig. 1), which exhibited parallel evolutionary pathways of change through time towards bone-cracking ecomorphologies [10]. These axes are relative skull width (width-to-length ratio, W∶L) and relative skull depth (depth-to-length ratio, D∶L). Even though the actual evolutionary patterns of skull shape change is complex, the morphospace constructed in this study used only simple overall cranial dimensions; more sophisticated methods of generating theoretical skull shapes are actively being developed to better characterize the observed variation [54]. Nevertheless, simple variation along the axes used generates a two-fold difference in the functional attributes tested.

During the evolution of the hyaenid and borophagine canid lineages, species evolved from relatively long-snouted, shallow- and narrow-skulled forms to short-snouted, deep- and wide-skulled robust forms [10], [40], [41]. These general skull shape changes are associated with the increased biomechanical capability of the larger and more robust species to consume hard foods [43], [52], [55], [56]. The exact causal links between incremental morphological changes and functional improvements are not known (and therefore the morphospaces created here are hypothetical in nature), but mechanical functions of specific craniodental features in bone-cracking ecomorphologies have been proposed [37], [57], [58]. Among these features are the development of a domed forehead and enlarged masticatory muscles, which are manifested in relatively deeper and wider skulls, respectively [59]. Accordingly, hybrid morphospaces were created to encompass and extend this range of observed evolutionary trends. Both morphological parameters were altered from the plesiomorphic state seen in the skull of *Ictitherium* by (1) increasing dorsoventral skull depth relative to skull length, and (2) increasing lateral skull width in increments of 25% up to 200% deviation from the *Ictitherium* specimen. To examine skull functionality that fall below this area, relative width and depth ratios of 75% from *Ictitherium* were also examined.

**Figure 2:**
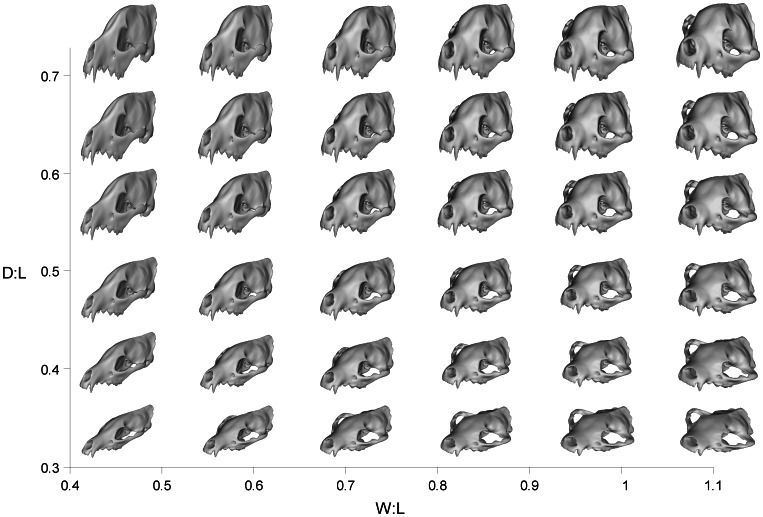
Theoretical models generated by geometric modification of an *Ictitherium* skull. The hybrid morphospace occupied by the 36 models spanned D∶L ratios from 0.33 to 0.73 and W∶L ratios from 0.42 to 1.11. Theoretical skull shapes are shown in rostral-lateral view.

The two main axes of cranial shape change formulated were used to form a two-dimensional morphospace analogous to Raup's [17] classic “cube” of geometric parameters of shell coiling (Fig. 2). The cranial parameters used here, however, do not constitute theoretical morphospace in the strict sense; the direction of change along each morphological axis were chosen for analysis based on previous work using empirical morphospace [10]. This type of morphospace might also be referred to as a “combined” morphospace utilizing elements of both theoretical and empirical morphospaces [19].

### Measures of function

Conventional adaptive landscapes rely conceptually on direct measures of survival and reproduction; such measures are dependent on environmental and ecological conditions at the specific temporal and spatial scale being examined [5], [16]. The creation of a functional landscape, as defined here, aimed to measure more universal features of craniodental systems based on biomechanics. Bite force, regardless of the means for its estimation in living and extinct organisms, is one parameter that is crucial for vertebrates in both prey apprehension and mastication [60], [61]. It is particularly important for bone-cracking ecomorphologies, as bite force is one direct determinant of the size of prey bone that can be consumed [39], [62]. Thus, the bite force performance of fossil and living carnivorans is expected to be of major importance.

Similarly, it has been argued that skull strain energy, a measure of the work done in deformation of an object in FEA simulations, is a suitable measure of functional efficiency [32]. This argument is based on the logic that biological objects (e.g. skulls) with maximum stiffness for a given volume of material (i.e. low strain energy during deformation) should be favored by selective processes that maximize functionality [32]. Skull strain energy is used as a second axis of function in this study. The skulls of species in bone-cracking lineages are expected to be selected for increased stiffness per amount of skull bone, in order to perform the intensive bone-cracking behavior which places large amounts of stress and strain on the skull.

### Functional landscape

Analogous to an adaptive landscape, where the third-dimension is a fitness axis used to document adaptive peaks and valleys over a bivariate plot of morphological parameters [8], a functional landscape charts functional properties measured by a biomechanical axis over the bivariate plot of morphological parameters. The functional properties of bite force and skull strain energy are distinguished from measures of fitness because the former measure relatively narrow aspects of mastication, and not the overall organismal fitness or performance. Nevertheless, for lineages such as hyaenids and borophagine canids that experienced directional evolution towards bone-cracking ecomorphologies, the two functional properties analyzed were probably important for bone-cracking performance.

The incremental changes in morphological parameters create hypothetical morphotypes that show variation along the same directions as observed empirically in hyaenids and canids (Fig. 2). The association between parameters of skull shape and the ecological habits of extant carnivorans has been demonstrated in empirical morphospaces created by geometric morphometrics analyses [63], [64]. Conceptually, the morphological parameters used can be supplemented with other functionally relevant parameters that are particular to the research question being addressed. Similarly, there may be other functional properties in addition to bite force and skull strain energy that are relevant to the specific type of functional morphology being examined. In its basic concept, the functional landscape is a functional manifestation of an adaptive landscape, its fitness axis (commonly the z-axis) having been modified to measure aspects of biomechanical function, which underlies organismal performance in relevant tasks.

### Generation of theoretical models

Theoretical morphotypes representing incremental deviation of the two morphological parameters were generated by modification of an *Ictitherium* digital skull model. A complete and intact skull of the late Miocene hyaenid *Ictitherium* sp. (HMV 0163, Hezheng Paleozoology Museum, Gansu Province, China) was scanned using computed tomography (CT) at Lanzhou University Hospital No. 1 (Gansu Province, China) with a Siemens Somatom Sensation 64 scanner (120 KV, 304.00 mAs); images had a pixel size of 0.2578 mm, resolution of 512×512 pixels, and 0.36 mm interslice distance. Data were exported in the DICOM (Digital Imaging and Communications in Medicine) format. The cranium and mandible of the specimen were separated and digitized using the software program Mimics 13 (Materialise NV). Digital reconstructions, including internal morphology, were exported in the stereolithography format (*.stl). The files were then imported into Geomagic Studio 10 (Geomagic, Inc.) where generation of theoretical morphotypes took place.

Skull depth and width in theoretical morphotypes were changed by scaling the original digital model of *Ictitherium* in the respective axes by a set percentage (75%–200% of original). The axes of change were aligned so that depth increased along the line connecting the carnassial tooth and the top of the frontal dome (Figs. 1–2); width increased along the axis perpendicular to the long axis of the skull. The modified theoretical morphotypes were then exported into Strand7 2.3.7 finite element analysis software program (G+D Computer Pty Ltd), where finite element meshes were generated.

The finite element meshes representing different morphotypes were modeled with identical forces, material properties, and boundary conditions. Because the fourth premolar (carnassial tooth) represents a synapomorphy of Carnivora for shearing and masticating meat, all models simulated unilateral bites with the carnassial. Although specialized bone-cracking hyaenids and certain borophagine canids (e.g. *Aelurodon*) evolved robust P3 as the main bone-cracking tooth, other specialized canids and less specialized hyaenids do not equally emphasize the robustness of this tooth. Therefore, the carnassial tooth simulation provided a common point of comparison across convergent specialist and generalized species. Furthermore, previous findings indicate that both P3 and P4 exhibit higher mechanical advantage in the extant spotted hyena compared to gray wolf [43], thus adaptive signals in P3 bone crackers would be recorded in P4 simulations as well. Three jaw-closing muscle groups were modeled: temporalis, masseter, and pterygoid. The relative contributions of the muscle groups to total input force were set at 67% (Temporalis), 22% (Masseter), and 11% (Pterygoid); these values were based on wet weight of the relative muscles in modern *Crocuta crocuta* [65]. Proportions of 64%, 22%, and 11% have been reported for canids [28], [66], [67]; the small differences between hyaenids and canids were assumed to be negligible for the model results studied, and the construction of models from actual specimens (including canids) used the first set of percentages for consistency. Muscle activation on the balancing (non-biting) side cranium was adjusted to 60% of the total input force on the working (biting) side cranium; ratios across the muscle groups remained the same [68]. Force vectors within each muscle attachment area were divided evenly over the entire area, with adjustment for wrapping of musculature around the cranial muscle attachment sites using the Boneload program [69]. Muscle force vectors in the respective muscle groups were oriented toward centroids of each muscle group at the attachment sites on the corresponding dentaries. A gape of 30 degrees was simulated for all models, close to the optimal angle found in *Canis lupus dingo* [70]. A total of 39,820 N of input muscle force was simulated in all models, and the output bite force was calculated as mechanical advantage (output force/input force) with a maximum range of 0.0 (no output force) to 1.0 (output force = input force). All models were also adjusted to have identical total surface areas (1×10^6^ mm^2^), to allow comparison of performance variables among theoretical morphotypes as a function of shape changes, but not size [32]. This particular ratio of input force (39,820 N) to surface area ratio (1×10^6^ mm^2^) matched the ratio used by Tseng and Wang [52], which was derived from the force-surface area ratio in their *Canis lupus* model that was validated by maximal measured bite force in *Canis familiaris* [71].

Three nodal constraints were placed on the cranium models: the left and right temporomandibular joints (TMJ), and the unilateral bite point. The bite point was modeled as a nodal constraint fixed from all translational and rotational movements. The TMJ was modeled as a single nodal constraint in the middle of each glenoid fossa, fixed from all but rotational movement in the sagittal plane. All models were given a single set of material properties, representing typical values for mammalian cortical bone. All analyses were linear and static, therefore only two material parameters were required: Young's (Elastic) modulus = 20 GPa, and Poisson's ratio = 0.3. Heterogeneous models that contain multiple material properties have been shown to have higher stresses and bite forces compared to identical models made with a single set of material properties; such differences in results are acknowledged, but they are assumed to have no great effect on the comparative context being pursued in this study [25], [52], [55].

**Figure 3:**
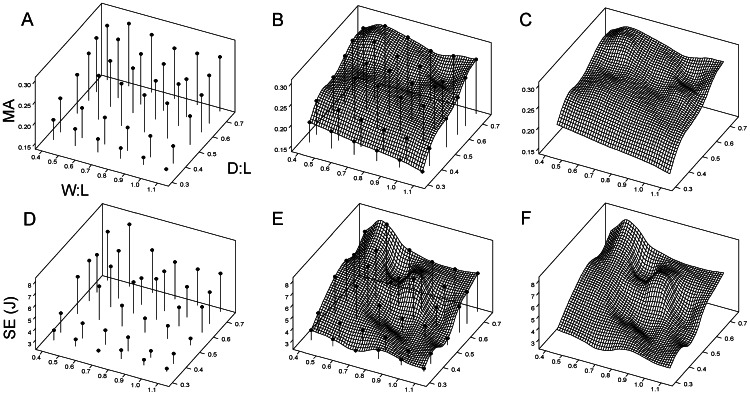
Construction of the functional landscape from theoretical morphologies. W∶L and D∶L are plotted on the x- and y-axes, respectively. The functional properties mechanical advantage (MA) and skull strain energy (SE, in joules) are plotted on the z-axis. A, D, three-dimensional plots of the data points from analysis of theoretical models; B, E, the wire frame mesh overlaid and interpolated using the theoretical models; C, F, the theoretical models removed, leaving the mesh representing the functional landscapes for MA and SE, respectively. For data values see Table S5.

Bite force output is graphed as mechanical advantage (MA) and skull strain energy (SE) values recorded in Joules. Both were plotted against bivariate plots of the two morphological parameters (D∶L and W∶L ratios) as wireframe plots, upon which simulation results from models of actual hyaenid and borophagine species were plotted (Fig. 3).

### Skull dimensions of actual species

To use the functional landscape in predictions of functional evolution in actual lineages, the W∶L and D∶L ratios of actual hyaenids and canid species were measured from specimen photos. The dataset of fossil and extant hyaenids and canids from Tseng and Wang [10] was used. Nine hyaenid species and 15 canid species were complete enough to be measured. Means were used where sample size >1, and specimens that were used in FEA (see below) were plotted individually (Table 1, S1, S2).

**Table 1:** List of actual models and species measurements of hyaenids and canids used in the study.

Actual models:	
Hyaenidae	Canidae
*Crocuta crocuta*	*Lycaon pictus*
*Parahyaena brunnea*	*Canis lupus*
*Ikelohyaena abronia* †	*Borophagus secundus* †
*Chasmaporthetes lunensis* †	*Epicyon haydeni* †
*Ictitherium sp.* †	*Microtomarctus conferta* †
*Proteles cristata*	*Mesocyon coryphaeus* †

Additional measurements:	
*Crocuta crocuta* (n = 45)	*Canis dirus* (n = 1)†
*Adcrocuta eximia* (n = 3)†	*Borophagus secundus* (n = 1)†
*Hyaena hyaena* (n = 1)	*Epicyon haydeni* (n = 1)†
*Hyaenictitherium wongi* (n = 1)†	*Epicyon saevus* (n = 1)†
*Proteles cristata* (n = 3)	*Aelurodon ferox* (n = 3)†
	*Aelurodon mcgrewi* (n = 1)†
Percrocutidae	*Aelurodon taxoides* (n = 1)†
*Dinocrocuta gigantea* (n = 3)†	*Protomarctus optatus* (n = 1)†
	*Phlaocyon leucosteus* (n = 1)†
	*Desmocyon matthewi* (n = 1)†
	*Paraenhydrocyon josephi* (n = 2)†
	*Hesperocyon gregarius* (n = 1)†

For list of specimen numbers see Tables S1, S2.

† extinct taxon.

### Models of actual species

FE models of actual fossil and living species of Hyaenidae and borophagine canids were constructed as described above for the theoretical models. Most of the models were existing ones taken from previous studies [43], [52], [55], [65]. Bite force and skull strain energy values were obtained from analyses after all models were standardized so that the ratios of total muscle input force to total model surface area were kept constant across all models [32]. Such standardization allowed the absolute size of models to be removed, and comparisons of skull shape and function measured. This type of comparisons are desired in this case because the functional landscape is constructed from morphological parameters that approximate evolutionary shape changes, most of which are not allometric in hyaenids and canids [10]. Also, body size increased dramatically over the course of evolution in the two carnivoran groups examined, so that bite force would show increases even in absence of biomechanical adaptations. Therefore, comparisons solely based on skull shape appeared to be the most appropriate.

To examine evolutionary trends predicted by the functional landscape, a series of FE models that represent different degrees of specialization for bone-cracking in the hyaenid and borophagine lineages, respectively, were used. The hyaenids *Proteles cristata* (J050607T02, ZJT comparative collection, prepared dry skull), *Ictitherium* sp. (HMV 0163), *Chasmaporthetes lunensis* [55], [72], *Ikelohyaena abronia* [65], *Parahyaena brunnea* (MVZ 117842, Museum of Vertebrate Zoology, University of California, Berkeley), and *Crocuta crocuta* [55] were analyzed. The fossil and modern canids analyzed included *Mesocyon coryphaeus*, *Microtomarctus conferta*, *Epicyon haydeni*, *Borophagus secundus*, and *Canis lupus* from Tseng and Wang [52], and *Lycaon pictus* from Tseng and Stynder [65]. In addition, the percrocutid *Dinocrocuta gigantea*, a feliform carnivoran that convergently evolved bone-cracking morphology independent of hyaenids or canids, was included in the analysis using the model from Tseng [43]. All specimens, except for *P. cristata*, are deposited in the museum collections listed above and described in the relevant publications cited. The raw CT data for *P. cristata* are archived online in Dryad (doi:10.5061/dryad.r2b1h). A total of 13 models of actual fossil and extant species were used.

In addition to MA and SE values, the stress distributions on the skulls of actual species were also visualized. Values of von Mises stress, which approximate materials that fail under a ductile mode of fracture, were used [26], [73]. As FEA conducted on models of actual species were scaled in a similar manner to the theoretical models, the distribution of von Mises stress on the skull represents relative levels of stress that can be directly compared across species. High levels of stress under such comparisons can therefore be interpreted as likely areas of material failure.

## Methods

The hybrid morphospace comprised 36 theoretical models, onto which a wire mesh was interpolated to create the functional landscapes (Figs. 2–3). Two separate landscapes were created, one for mechanical advantage (MA; Fig. 3A–C) and the other for skull strain energy (SE; Fig. 3D–F). The landscapes showed predictable trends of variation. Increasing skull depth, regardless of the starting skull width, translated into higher MA and higher SE (Fig. 3). Increasing skull width generated progressively lower MA and SE at shallower skull depths, but the patterns became more complex at higher skull depths (Fig. 3). Peaks in MA are found at skull depth-to-length (D∶L) ratio of >0.7 and width-to-length (W∶L) ratios of 0.4–0.7 (Fig. 3A–C). Lowest MA values are found at D∶L<0.4 and W∶L>0.7 (Fig. 3A–C).

**Figure 4:**
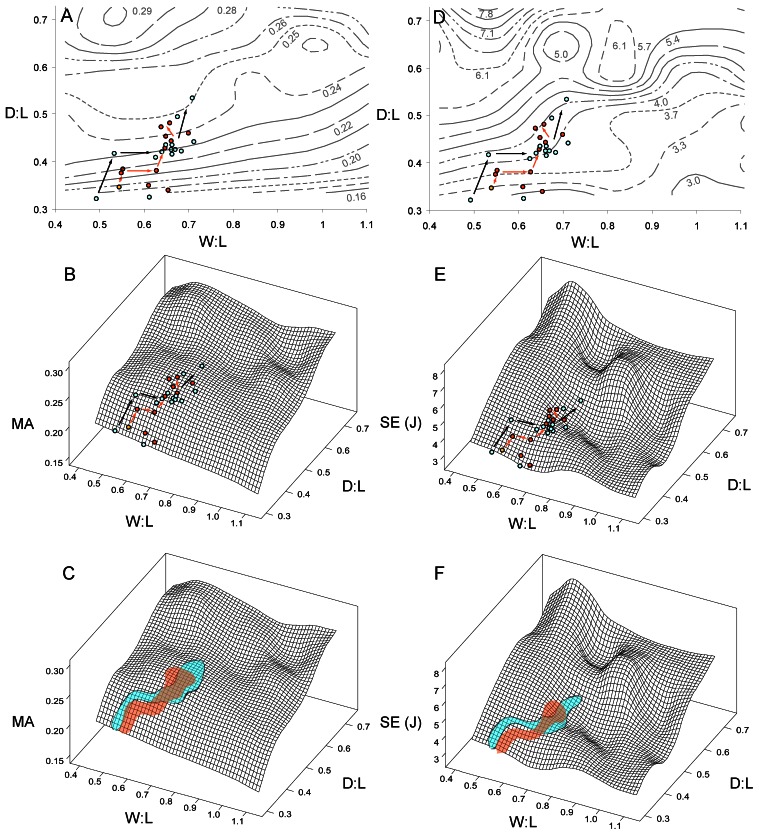
Distribution of actual species on the functional landscapes. A, D, distribution of hyaenids (dark circles) and fossil canids (light circles) on two-dimensional contour plots of MA and SE, respectively. Lines are isoclines. B, E, distribution of hyaenid and canid species on the three-dimensional functional landscapes for MA and SE, respectively. C, F, the pathways occupied by the hyaenid (shaded) and canid (outlined) lineages on the MA and SE landscapes, respectively. Sequential arrows indicate directions of change from less derived, earlier species to more derived, younger species. Note continuous climb on the MA landscape and shifting towards shallower slopes on the SE landscape. For data values of species models see Table S6.

**Figure 5:**
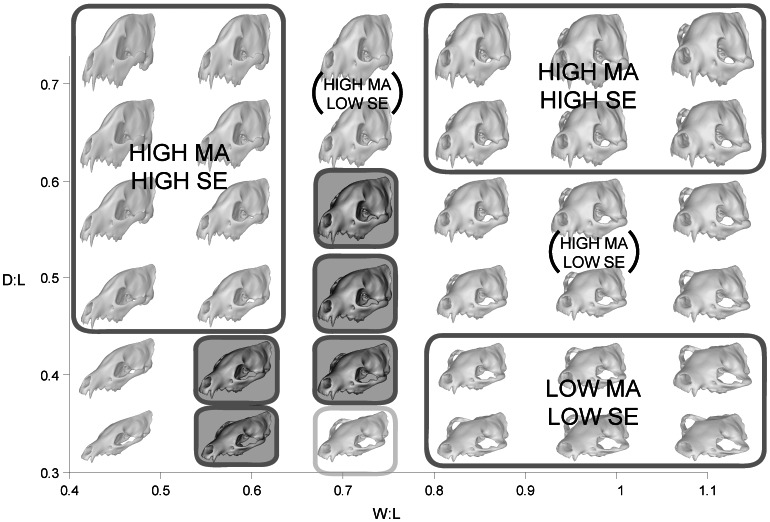
Locations of optimal functional capability in the hybrid morphospace. Small shaded squares represent theoretical models matched by existing hyaenid and canid species (small unshaded square shows position of insectivorous *Proteles cristata*). Suboptimal regions are shown in larger squares. Regions marked by parenthesized labels represent optimal areas not occupied by actual species.

D∶L and W∶L ratios of actual hyaenid and borophagine canid species overlapped extensively in their distribution on the functional landscape (Fig. 4). Furthermore, the species followed an evolutionary pathway from D∶L 0.3–0.4 and W∶L 0.5–0.6 to D∶L ∼0.5 and W∶L ∼0.7 (Fig. 4A, D). This pathway showed a continuous climb towards higher elevation on the MA landscape (Fig. 4B), and a path into an adaptive valley on the SE landscape (Figs. 4E). The pathways occupied by actual hyaenids and canids traversed a region of increasing MA and moderately low SE, which is bordered at the bottom right with a large region of low MA and low SE theoretical shapes (Fig. 5). In the upper regions are high MA and high SE shapes; both of these regions represent suboptimal areas (Fig. 5).

**Figure 6:**
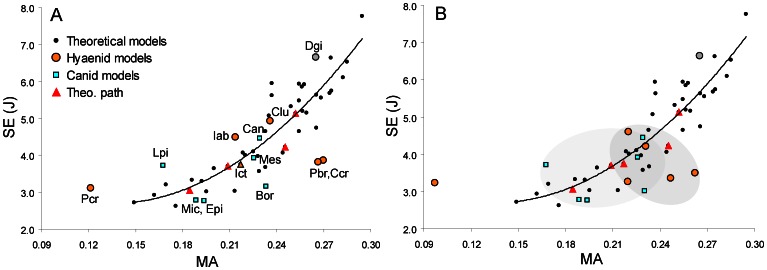
Theoretical and actual MA and SE values. A. distribution of theoretical models overlaid with values from FE models of actual species, all scaled by total surface area. B, distribution of theoretical and actual models, the latter scaled by condylobasal length of the skull. Red triangles indicate the theoretical pathway traveled by actual species on the functional landscape. The positions of hyaenid (darker shade) and canid (lighter shade) groupings are shown as ovals in (B). Species abbreviations (hyaenids): Ccr, *Crocuta crocuta*; Hlu, *Chasmaporthetes lunensis*; Iab, *Ikelohyaena abronia*; Ict, *Ictitherium sp.*; Pbr, *Parahyaena brunnea*; Pcr, *Proteles cristata*. Canids: Bor, *Borophagus secundus*; Can, *Canis lupus*; Epi, *Epicyon haydeni*; Lpi, *Lycaon pictus*; Mes, *Mesocyon coryphaeus*; Mic, *Microtomarctus conferta*. Percrocutid: Dgi, *Dinocrocuta gigantea*.

The regions of the MA vs. SE plot occupied by actual species represent a pathway from low MA (∼0.18) towards higher MA (∼0.25) at or below the fitted curve (SE = 174.74*MA^2^−47.04*MA+5.8609, r^2^ = 0.8363) for the theoretical models (Fig. 6). The MA values of models of actual species covered a slightly larger range than predicted by theoretical models, from ∼0.16 to ∼0.27 (Fig. 6A). The exception is the myrmecophagous hyaenid *Proteles cristata*, which has an MA of ∼0.12, lower than all actual species and theoretical models. SE values of actual species followed the overall trend predicted by the theoretical models, but do not follow the theoretical pathways exactly. To test for potential differences created by scaling factor, models of actual species were re-analyzed after scaling by total volume, total muscle attachment surface area, or total skull length (condylobasal length). Volume- and muscle-scaled models returned essentially identical results as the total surface area method (Fig. 6A). Scaling by skull length returned similar results, except that MA values for the derived hyaenids *Crocuta crocuta* and *Parahyaena brunnea* were lower, and SE values for *Ictitherium* and *Chasmaporthetes lunensis* were also lower (Fig. 6B). Such differences did not change the overall trends, however.

**Figure 7:**
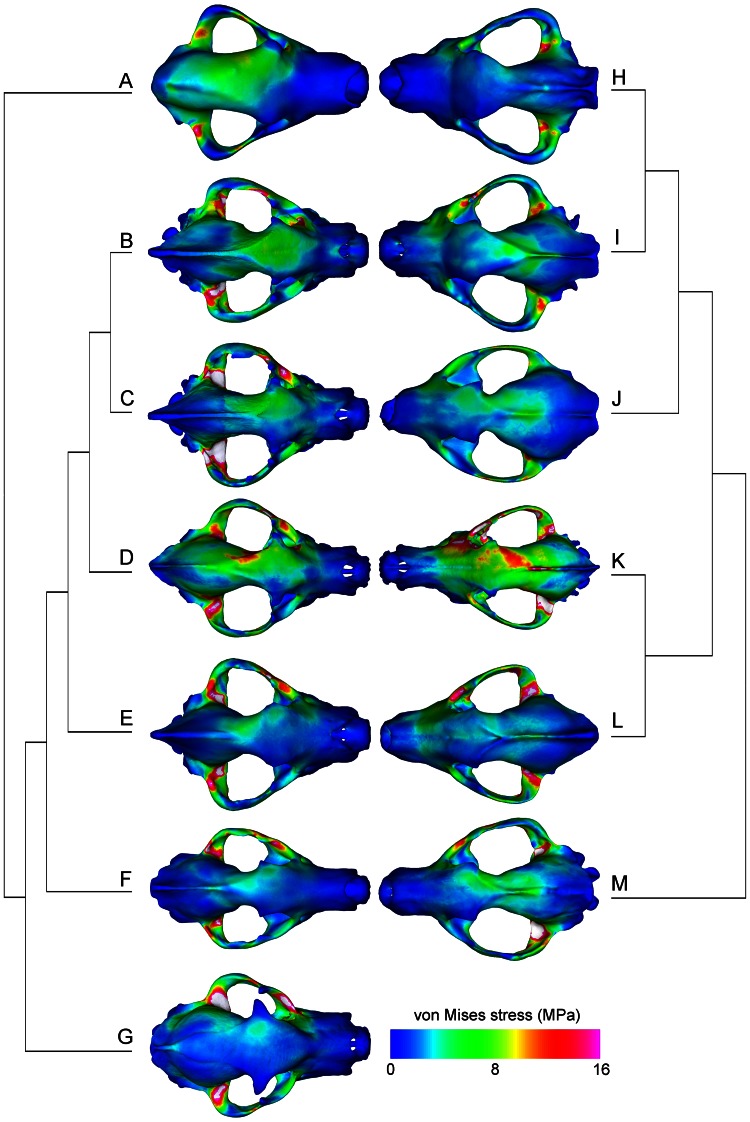
Stress distributions on the FE skull models of actual fossil and extant species. A, *Dinocrocuta gigantea*; B, *Crocuta crocuta*; C, *Parahyaena brunnea*; D, *Ikelohyaena abronia*; E, *Chasmaporthetes lunensis*; F, *Ictitherium sp.*; G, *Proteles cristata*; H, *Epicyon haydeni*; I, *Borophagus secundus*; J, *Microtomarctus conferta*; K, *Canis lupus*; L, *Lycaon pictus*; M, *Mesocyon coryphaeus*. Phylogenetic relationships for hyaenids (A–G) based on Werdelin and Solounias (1991), and for canids (H–M) based on Wang (1994), Wang et al. (1999), and Tedford et al. (2009).

Von Mises stress distributions on the actual models showed a general trend of increasingly stressed fronto-parietal regions in hyaenids (Fig. 7A–G). The canid models showed no such trend, and in general had moderate levels of von Mises stress spread over the dorsal cranium, except for elevated stress levels in *Canis lupus* and decreased levels in *Epicyon haydeni* (Fig. 7H–M). The fronto-parietal region in *Ikelohyaena abronia* and *Canis lupus* showed the highest peak stress, and in all models the temporomandibular joints tend to have elevated stress levels (Fig. 7).

## Discussion

The generation of simplified functional landscapes was used to test the hypothesis that convergent morphological evolution in canids and hyaenids can be explained in terms of functional evolution towards more optimal bone-cracking capability. Theoretical skull shapes showed a general increase in mechanical advantage (MA) with higher D∶L ratios, although the narrower (lower W∶L) skulls had largest levels of strain energy (Fig. 3). Actual hyaenid and canid species showed a steady climb up the MA landscape, at the same time moving along topological isoclines in SE. Such a pattern of evolution is consistent with optimization theory; in this case two functions of the skull, maximizing MA and minimizing SE, are optimized by traveling upslope on the MA landscape and moving along topological isoclines on the SE landscape (Fig. 4). Therefore, the hypothesis that convergent morphologies shared convergent functional capability is supported.

Hyaenid and canid models are adjacent to each other on the MA vs. SE plots, with more derived species having higher MA (Fig. 6). As predicted by the landscape model, the path from less derived species to more specialized species tend to occur downward (i.e. smaller SE for a given MA) or rightward (i.e. larger MA for a given SE) on the plot (Fig. 6). Such distribution is expected under an optimization model. The functional correlation of the MA and SE distributions is further supported by the fact that *Proteles cristata*, a specialized insectivorous hyaenid, does not crack bones, and accordingly has very low MA towards the bottom left corner of the MA vs. SE plot (Fig. 6). This exception also supports the interpretation that there is an adaptive coupling of functional attributes and ecology in this sample of carnivorans.

Overall, among the theoretical models used to construct the functional landscape, only a small number overlapped with actual morphologies (Fig. 5). In the upper left and upper right regions high MA is coupled with high SE, making those morphologies sub-optimal; those areas are accordingly not occupied by actual species (Fig. 5). The bottom right corner is marked by both low MA and low SE, and is similarly not optimal. The actual path taken by canids and hyaenids constitutes a route of increasing MA at relatively small cost in SE increase (Fig. 5). Therefore, the functional landscape distributions of actual species predict skull MA to be maximized relative to increase in skull SE through evolution. The distribution of MA versus SE values for the 13 actual skull models show an intermediate position within the range of theoretical morphologies, overlapping the regions predicted by the functional landscape, therefore supporting theoretical predictions (Fig. 6).

**Figure 8:**
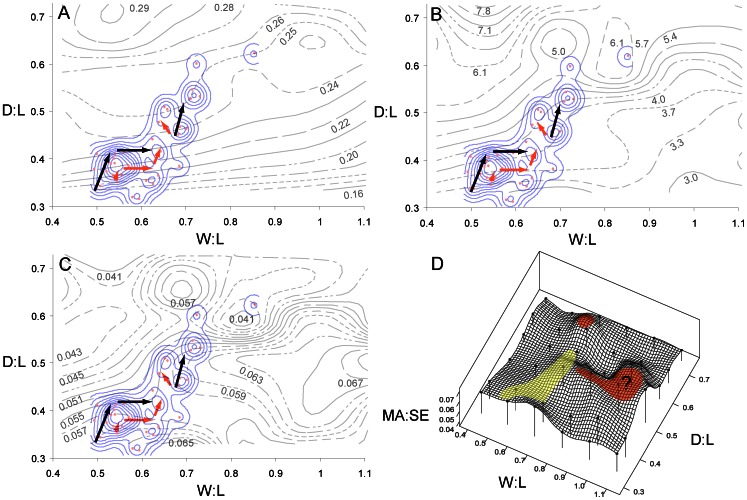
Distributions of modern North American and East African carnivoran species on the functional landscapes. Distributions are plotted on the MA (A), SE (B), and MA∶SE (C–D) landscapes. Arrows indicate pathways of evolution for hyaenids (light arrows) and borophagine canids (dark arrows). Species distributions of modern carnivoran species are plotted as solid contours. Peaks on the MA∶SE landscape (D) represent optimized theoretical skull shapes that are either realized (light shade) or unoccupied (dark shade, with question mark). (D) corresponds with Figure 5.

The remaining unoccupied regions in the functional landscape, however, indicate that the hypothesis predicting the derived morphotypes occupying functional optima on the landscape was not supported. Contrary to expectation, the most optimized theoretical shapes in the functional landscape are not occupied by actual species (Fig. 5). Skull shapes with D∶L = 0.7, W∶L = 0.7 and D∶L = 0.5, W∶L = 0.8–1.1 tend to have high MA and relatively low SE, making them more suitable for generating large bite forces than shapes toward the central and bottom left regions of the landscape, where canids and hyaenids are located (Fig. 5). There appears to be no visible barriers or functional valleys on the MA landscape, or prohibitively high SE peaks on the SE landscape, to explain the lack of actual species in those regions (Fig. 4). To check whether this bias in distribution is a function of similarly restricted skull shape changes specific to hyaenids and borophagine canids, the modern carnivoran dataset composed of 37 North American and East African carnivoran species from Tseng and Wang [10] was plotted onto the functional landscapes (Fig. 8, Table 2, S3, S4). The pathways taken by canid and hyaenids species overlapped with the skull shapes observed among the major representatives of modern carnivoran families (Fig. 8A–C). Only the cheetah, *Acinonyx jubatus*, was distinct from all other carnivorans by much higher W∶L ratios that placed the species on an SE peak (Fig. 8).

**Table 2:** List of modern North American and East Africa carnivoran species used to construct contour of carnivoran distribution.

North America	n	East Africa	n
*Alopex lagopus*	10	*Acinonyx jubatus*	2
*Canis latrans*	10	*Atilax paludinosus*	5
*Canis lupus*	11	*Bdeogale crassicauda*	5
*Gulo gulo*	5	*Canis aureus*	8
*Lynx canadensis*	5	*Caracal caracal*	1
*Lynx rufus*	4	*Civettictis civetta*	4
*Martes pennanti*	9	*Crocuta crocuta*	45
*Mephitis mephitis*	4	*Felis sylvestris*	7
*Mustela frenata*	7	*Genetta rubiginosa*	13
*Neovison vison*	2	*Herpestes sanguineus*	8
*Procyon lotor*	3	*Hyaena hyaena*	1
*Puma concolor*	9	*Ichneumia albicauda*	2
*Taxidea taxus*	6	*Ictonyx striatus*	3
*Urocyon cinereoargenteus*	1	*Lycaon pictus*	8
*Ursus americanus*	10	*Mellivora capensis*	1
*Ursus arctos*	9	*Nandinia binotata*	5
*Vulpes vulpes*	11	*Otocyon megalotis*	7
		*Panthera leo*	24
		*Panthera pardus*	7
		*Proteles cristata*	3

For specimen numbers see Tables S3, S4 and Tseng and Wang [10].

The extensive overlap of modern carnivorans with the evolutionary sequence of canids and hyaenids indicates constraint in skull shape disparity within the hybrid morphospace of theoretical possible shapes (Fig. 2). Presence of such a higher-level constraint created a limitation on the evolution of functionally more optimized skull shapes in bone-cracking carnivorans (Fig. 8). Of course, the complex suite of functions that the mammalian skull plays in mastication, food acquisition, and sensory reception meant that constraints on the realized skull shapes are more complex than just biomechanical ones. In the absence of additional non-biomechanical constraints, one optimal path to maximize MA and minimize SE would be to travel along topological lines at D∶L between 0.3 and 0.4 towards higher W∶L ratios (Fig. 8C–D). At higher W∶L ratios, SE increases more slowly and therefore those shapes have relatively higher MA. In reality, there appears to be a constraint in increasing W∶L ratio across the modern carnivorans analyzed, and consequently skull shape evolved towards higher D∶L with an upper limit of W∶L ∼0.7 to instead increase MA on local (but not global) optima (Fig. 8).

The high-MA high-SE skull of *Dinocrocuta gigantea* also demonstrates the presence of others factors in determining performance in addition to the two functional properties examined. The largest bone-cracking carnivorans examined in this study, *Epicyon haydeni* and *Dinocrocuta gigantea*, share similarities in skull shape but not in biomechanical attributes (Fig. 7). *Epicyon* has a low-MA and low-SE skull, in contrast to the high-MA high-SE skull of *Dinocrocuta*. This seemingly contradictory result might be explained by the one-to-one form to function property of mechanical advantage [16]. Mechanical advantage by itself is a scale-free measure of force generation, but in fact a system with high MA and low absolute muscle force can generate the same resulting bite force as a system with low MA and large absolute bite force. Therefore, the disparate distributions of *Dinocrocuta* and *Epicyon* can in fact represent similar performing morphologies that converge along another axis of evolutionary change, namely body size. A large body size would allow *Epicyon* to generate bite forces required to crack bones to a comparable degree as smaller, more shape-adapted skulls of *Crocuta* and *Borophagus*. On the other hand, the large body size of *Dinocrocuta* would allow a smaller muscle input to generate sufficient bone-cracking bite forces, therefore not producing the high-SE predicted at its maximum capability (Fig. 6). Such alternatives to evolutionary changes in skull shape can be further coupled with behavior, in which bones of smaller prey are cracked and consumed, and bones of larger prey intentionally left alone. With this interpretation, body size increase in bone-cracking carnivorans as a masticatory adaptation would be analogous to larger body size in ungulates as a defense mechanism, in that both increases in body size alone constitutes an adaptation. Whether “body-size” specialists should constitute a distinct sub-category of bone-cracking ecomorphology is a fascinating issue that remains to be explored. Archaic mammals such as creodonts and condylarths, for example, evolved dental morphology and body size approaching the larger carnivoran bone-crackers, even though the skulls of most creodonts do not share the suite of morphological features seen in carnivorans [35].

A concept intimately associated with adaptive landscapes is the macroevolutionary ratchet, which has been studied in carnivorans [53], [74]. The limited number of alternative means of morphological specialization is associated with decrease in morphological disparity in repeatedly specialized lineages, which affected the long-term fitness of those lineages [34], [53]. In this context, generalist species are located at lower elevations of the adaptive landscape, and specialists are higher up adaptive peaks; the macroevolutionary ratchet can be visualized as the evolutionary process of moving up in elevation on the landscape [75]. Catastrophic, sometimes even localized, events may shift the position of those adaptive peaks, causing the demise of specialists by their very inability to move or survive in other regions of the fitness landscape [75]. Others argue for the mobility and dynamic nature of adaptive peaks through time, which may imply a different mode of adaptation and specialization of organisms that involves more evolutionary “adjustment” to current peaks [8]. The fact that convergent canids and hyaenids evolved via pathways within the overall distribution of modern carnivorans indicates that a general constraint on skull depth and width ratios is present, perhaps as a more general phenomenon than caused by specific factors in a macroevolutionary ratchet model for bone-cracking specialists (Fig. 8). Nevertheless, it would be interesting to further explore whether the pathways on the functional landscape are “one-way streets”, and if the distance already traveled by a particular lineage may indeed represent the macroevolutionary ratchet in action.

The proxy for functionality used in this study, namely measures of bite force and skull strain energy, are biomechanical function indicators, arguably not a very complete measure of fitness (using a definition of the organismal ability to both survive and reproduce). However, the fact that terminal members of the lineages studied represent the best examples of *Crocuta*-equivalent bone-cracking ecomorphologies in the Cenozoic, and that their evolutionary processes show overwhelming trend towards robust craniodental features, suggest that in this case the functional properties likely would have been quite important in their evolution. Furthermore, carnassial mechanical advantage is a common selective parameter for all carnivorans, and measures of its biomechanical function are directly linked to mastication and food intake. One can also argue that plotting phylogenetic trends onto the static functional landscape is not greatly affected by the possibility of shifting adaptive peaks in other types of landscapes which are contingent upon environmental variations [8]; biomechanical function underlies the capability of different species to utilize harder food, which existed in the form of prey skeletal remains regardless of their taxonomic identity or the surrounding environment. In other words, the same selective pressures for masticatory capability would exist independently of environmental changes, as long as larger vertebrate prey are present. Thus, performance measures based on physical principles such as mechanical advantage are suitable rulers to test specific form-function hypotheses in ecomorphological contexts.

Regardless of the simplicity of a two-dimensional framework, the resulting distribution of actual species on the functional landscape shows a remarkable consistency of maintaining MA∶SE ratios throughout the region occupied by bone-cracking canids, hyaenids, and the corresponding modern faunas (Fig. 8). Such a pattern indicates an overarching selection for the maintenance of strong skulls and efficient bites across Carnivora, attributes which are principal in both active hunting and passive scavenging behaviors. Despite the outstanding morphological features of the skull and teeth in specialized bone-cracking ecomorphologies, the functional properties of those derived ecomorphs still operated within the bounds of the carnivoran distribution. Again, the notable exception in the modern east African fauna is the cheetah, *Acinonyx jubatus*. Skull shape in the cheetah has fallen off the tall ridges on the functional landscape, and is located in a valley with low MA. The strict requirements for speed may have overridden the base functional demands of mastication, demonstrating that such deviation from the major trend is nevertheless feasible (Fig. 8).

Among metazoan animals, redundancy in body segments has been proposed to enhance evolutionary potential for differentiation in functions [16]. An analogous explanation can be applied to the plesiomorphically homodont dentition of vertebrates, which evolved into highly heterodont dentition in mammals. Carnivores exhibit fine examples of diversified function of heterodont teeth [76]. The shallow, slashing bites of pursuit predators are made using the anterior incisors and canines, and the crushing bites of omnivores are made with the posterior bunodont molars [77]. Such differentiation in dental function is shared by all carnivorans on a more general level, indicating the presence of multiple axes of functional properties for different tooth positions alone. Carnassial function, the focus of the current study, for example, should be supplemented with study of functional properties in other teeth in order to more fully characterize the potential selective forces that shape craniodental morphology. Such integration requires more complex mathematical formulations of a multi-dimensional problem, of which the current study represents a two-dimensional first step that is easily visualized.

The fact that the functional landscapes predicted movement of canid and hyaenid species through a more or less isoclinal ridge in the D∶L and W∶L morphospace of MA∶SE ratios suggests there are other important factors besides general skull dimensions in the functional evolution of bone-cracking ecomorphologies. Movement on the landscape towards deeper and wider skulls also allows more masticatory musculature to be present in the parietal region, which was not adjusted in the theoretical shapes analyzed here. In addition, the relative proportions of the rostrum and the braincase, and also the positions of the dentition relative to the masticatory muscles both affect mechanical advantage. Such changes require more sophisticated theoretical models, and more fine-tuned variations in FE skull models, which might be generated using algorithms derived from geometric morphometrics analysis [78]. Nevertheless, the usage of simple morphological parameters to create theoretical skull shapes was shown to be informative in discovering potential biomechanical and non-biomechanical constraints on overall skull shape in convergent evolution of adaptive morphologies in carnivorous mammals. Many more studies are needed to explore the begging questions and to improve the completeness of such theoretical frameworks.

In sum, a functional landscape framework constructed from theoretical morphologies showed the presence of functional peaks that are not attained by actual species. The pathways that actual species traversed, however, were nevertheless local optima of relatively high mechanical advantage and moderate skull strain energy. Predictions from the functional landscape are supported by results obtained using models of actual species, showing a clear link between form and function in the evolution of bone-cracking ecomorphologies. The restricted region occupied by a wider sampling of modern carnivorans on the functional landscape indicates higher-level phylogenetic constraint as an explanation for the unoccupied optimal peaks. The combination of theoretical morphology and functional modeling with FEA has been shown to be an informative approach to test adaptive hypotheses regarding morphological convergence, and has implications for applications in broader taxonomic contexts.

## Conclusions

An analytical framework combining biomechanical analysis of three-dimensional theoretical morphologies and functional landscapes to evaluate the evolutionary trends in actual lineages represents a novel approach to the study of convergent evolution. Modeling approaches such as finite element analysis not only permits the incorporation of fossil species into biomechanical simulations, but also provides comparative data that inform the robustness of previously hypothesized form-function relationships. Given an asymmetrical understanding of morphological disparity relative to its functional significance, many more such studies are needed, especially for extinct lineages. The case study of convergent, bone-cracking hypercarnivores showed that both biomechanical and broader-scale factors act to shape the observed morphologies of hyaenids and dogs, and that the existing morphological disparity in those two lineages likely represent only local optima in functional morphology. More sophisticated theoretical and functional frameworks will continue to shed light on mechanisms that underlie such prominent examples of evolutionary convergence.

## Supporting Information

Table S1 **Cranium ratio measurements of fossil canids.** Institutional abbreviations: AMNH, American Museum of Natural History, New York; F:AM, Frick Collection, American Museum of Natural History, New York; HMV, Hezheng Paleozoology Museum, Gansu, China; IVPP, Institute of Vertebrate Paleontology and Paleoanthropology, Beijing, China; LACM, Natural History Museum of Los Angeles County, California; MCZ, Museum of Comparative Zoology, Harvard University, Massachusetts; MVZ, Museum of Vertebrate Zoology, University of California, California; PPHM, Plains-Panhandle Museum, Texas; UAMZ, University of Alberta Museum of Zoology, Alberta, Canada; UCMP; University of California Museum of Paleontology, Berkeley, California. Other Abbreviations: D∶L, skull depth to length ratio; W∶L, skull width to length ratio.
(DOC)

Table S2 **Cranium ratio measurements of fossil hyaenids and percrocutids.** For abbreviations see Table S1 legend.
(DOC)

Table S3 **Cranium ratio measurements of extant North American carnivorans.** For abbreviations see Table S1 legend.
(DOC)

Table S4 **Cranium ratio measurements of extant east African carnivorans.** For abbreviations see Table S1 legend.
(DOC)

Table S5 **Theoretical models and their parameters.** D∶L, skull depth to length ratio; W∶L, skull width to length ratio; elements: number of four-noded tetrahedral finite elements in model; SE, skull strain energy (in Joules); adjSE, strain energy adjusted by model volume; Fout, output bite force (in Newtons); MA, mechanical advantage; S.T., solution time required for FEA (in minutes). Model files are deposited in Dryad (doi:10.5061/dryad.r2b1h).
(DOC)

Table S6 **Finite element model parameters of actual fossil and extant carnivorans analyzed in the study.** The number of elements were kept as close to 1,000,000 elements as possible for both actual and theoretical models. Three models had lower counts of elements (*P. brunnea, P. cristata, C. lupus*), but no correlation of MA or SE values to lower element counts was detected. Abbreviations as in Table S5. Model files are deposited in Dryad (doi:10.5061/dryad.r2b1h).
(DOC)
