# Supplementary material for: Testing Adaptive Hypotheses of Convergence with Functional Landscapes: A Case Study of Bone-Cracking Hypercarnivores
Source: PLoS One. 2013 May 29;8(5):e65305. doi: 10.1371/journal.pone.0065305 (PMC3667121; doi:10.1371/journal.pone.0065305)
Supplement: Table S1 — Cranium ratio measurements of fossil canids. Institutional abbreviations: AMNH, American Museum of Natural History, New York; F:AM, Frick Collection, American Museum of Natural History, New York; HMV, Hezheng Paleozoology Museum, Gansu, China; IVPP, Institute of Vertebrate Paleontology and Paleoanthropology, Beijing, China; LACM, Natural History Museum of Los Angeles County, California; MCZ, Museum of Comparative Zoology, Harvard University, Massachusetts; MVZ, Museum of Vertebrate Zoology, University of California, California; PPHM, Plains-Panhandle Museum, Texas; UAMZ, University of Alberta Museum of Zoology, Alberta, Canada; UCMP; University of California Museum of Paleontology, Berkeley, California. Other Abbreviations: D∶L, skull depth to length ratio; W∶L, skull width to length ratio. (DOC) [file pone.0065305.s001.doc]

**Table S1. Cranium ratio measurements of fossil canids.** Institutional abbreviations: AMNH, American Museum of Natural History, New York; F:AM, Frick Collection, American Museum of Natural History, New York; HMV, Hezheng Paleozoology Museum, Gansu, China; IVPP, Institute of Vertebrate Paleontology and Paleoanthropology, Beijing, China; LACM, Natural History Museum of Los Angeles County, California; MCZ, Museum of Comparative Zoology, Harvard University, Massachusetts; MVZ, Museum of Vertebrate Zoology, University of California, California; PPHM, Plains-Panhandle Museum, Texas; UAMZ, University of Alberta Museum of Zoology, Alberta, Canada; UCMP; University of California Museum of Paleontology, Berkeley, California. Other Abbreviations: D:L, skull depth to length ratio; W:L, skull width to length ratio.

| Taxon | Specimen # | W:L | D:L |
| --- | --- | --- | --- |
| *Aelurodon ferox* | F:AM 27346 | 0.66 | 0.43 |
| *Aelurodon ferox* | F:AM 61746 | 0.66 | 0.43 |
| *Aelurodon ferox* | F:AM 61757 | 0.64 | 0.42 |
| *Aelurodon mcgrewi* | F:AM 61778 | 0.67 | 0.43 |
| *Aelurodon taxoides* | F:AM 61781 | 0.63 | 0.41 |
| *Borophagus secundus* | F:AM 61640 | 0.71 | 0.53 |
| *Borophagus secundus* | UCMP 30101 | 0.68 | 0.49 |
| *Canis dirus* | LACM 2077 | 0.59 | 0.44 |
| *Desmocyon matthewi* | AMNH 49177 | 0.59 | 0.38 |
| *Epicyon haydeni* | PPHM 1100 | 0.68 | 0.42 |
| *Epicyon saevus* | F:AM 8305 | 0.65 | 0.43 |
| *Hesperocyon gregarius* | LACM-CIT 621 | 0.61 | 0.33 |
| *Hesperocyon gregarius* | UCMP 65380 | 0.49 | 0.32 |
| *Mesocyon coryphaeus* | NPS Joda-3348 | 0.62 | 0.35 |
| *Microtomarctus conferta* | LACM-CIT 1229 | 0.66 | 0.42 |
| *Paraenhydrocyon josephi* | F:AM 54115 | 0.62 | 0.31 |
| *Paraenhydrocyon josephi* | MCZ 2102 | 0.58 | 0.39 |
| *Phlaocyon leucosteus* | F:AM 8768 | 0.72 | 0.42 |
| *Protomarctus optatus* | F:AM 61156 | 0.53 | 0.42 |
