# Supplementary material for: Testing Adaptive Hypotheses of Convergence with Functional Landscapes: A Case Study of Bone-Cracking Hypercarnivores
Source: PLoS One. 2013 May 29;8(5):e65305. doi: 10.1371/journal.pone.0065305 (PMC3667121; doi:10.1371/journal.pone.0065305)
Supplement: Table S2 — Cranium ratio measurements of fossil hyaenids and percrocutids. For abbreviations see Table S1 legend. (DOC) [file pone.0065305.s002.doc]

**Table S2. Cranium ratio measurements of fossil hyaenids and percrocutids.**

| Taxon | Specimen # | W:L | D:L |
| --- | --- | --- | --- |
| *Adcrocuta eximia* | F:AM 28-L233 | 0.65 | 0.47 |
| *Adcrocuta eximia* | F:AM 35-B216 | 0.63 | 0.45 |
| *Adcrocuta eximia* | F:AM 41-L339 | 0.66 | 0.44 |
| *Chasmaporthetes lunensis* | MNCN-67100 | 0.66 | 0.48 |
| *Dinocrocuta gigantea* | HMV M0358 | 0.98 | 0.59 |
| *Dinocrocuta gigantea* | HMV X0361 | 0.70 | 0.52 |
| *Dinocrocuta gigantea* | IVPP V15649 | 0.76 | 0.45 |
| *Hyaenictitherium wongi* | AMNH 23032 | 0.55 | 0.38 |
| *Ictitherium sp.* | HMV 0163 | 0.55 | 0.38 |
| *Ikelohyaena abronia* | SAM-PQL 14186 | 0.63 | 0.38 |
