# Supplementary material for: Testing Adaptive Hypotheses of Convergence with Functional Landscapes: A Case Study of Bone-Cracking Hypercarnivores
Source: PLoS One. 2013 May 29;8(5):e65305. doi: 10.1371/journal.pone.0065305 (PMC3667121; doi:10.1371/journal.pone.0065305)
Supplement: Table S3 — Cranium ratio measurements of extant North American carnivorans. For abbreviations see Table S1 legend. (DOC) [file pone.0065305.s003.doc]

**Table S3. Cranium ratio measurements of extant North American carnivorans.**

| Taxon | Specimen # | W:L | D:L |
| --- | --- | --- | --- |
| *Alopex lagopus* | UAMZ 3155 | 0.56 | 0.52 |
| *Alopex lagopus* | UAMZ 5922 | 0.57 | 0.52 |
| *Alopex lagopus* | UAMZ 6906 | 0.59 | 0.58 |
| *Alopex lagopus* | UAMZ 6907 | 0.56 | 0.49 |
| *Alopex lagopus* | UAMZ 6908 | 0.55 | 0.52 |
| *Alopex lagopus* | UAMZ 6912 | 0.56 | 0.56 |
| *Alopex lagopus* | UAMZ 6913 | 0.55 | 0.56 |
| *Alopex lagopus* | UAMZ 6914 | 0.56 | 0.53 |
| *Alopex lagopus* | UAMZ 6918 | 0.55 | 0.54 |
| *Alopex lagopus* | UAMZ 6924 | 0.56 | 0.50 |
| *Canis latrans* | UAMZ 2172 | 0.50 | 0.44 |
| *Canis latrans* | UAMZ 2173 | 0.52 | 0.48 |
| *Canis latrans* | UAMZ 2179 | 0.53 | 0.49 |
| *Canis latrans* | UAMZ 2415 | 0.51 | 0.42 |
| *Canis latrans* | UAMZ 2418 | 0.54 | 0.47 |
| *Canis latrans* | UAMZ 2419 | 0.52 | 0.48 |
| *Canis latrans* | UAMZ 2429 | 0.52 | 0.46 |
| *Canis latrans* | UAMZ 2440 | 0.51 | 0.45 |
| *Canis latrans* | UAMZ 2825 | 0.52 | 0.46 |
| *Canis latrans* | UAMZ 3307 | 0.54 | 0.48 |
| *Canis lupus* | LACM 23010 | 0.54 | 0.54 |
| *Canis lupus* | UAMZ 7807 | 0.53 | 0.51 |
| *Canis lupus* | UAMZ 7810 | 0.52 | 0.47 |
| *Canis lupus* | UAMZ 7811 | 0.55 | 0.50 |
| *Canis lupus* | UAMZ 7816 | 0.57 | 0.48 |
| *Canis lupus* | UAMZ 7821 | 0.52 | 0.50 |
| *Canis lupus* | UAMZ 7835 | 0.56 | 0.48 |
| *Canis lupus* | UAMZ 7836 | 0.53 | 0.46 |
| *Canis lupus* | UAMZ 7838 | 0.55 | 0.48 |
| *Canis lupus* | UAMZ 7843 | 0.53 | 0.52 |
| *Canis lupus* | UAMZ 7844 | 0.54 | 0.49 |
| *Gulo gulo* | UAMZ 1147 | 0.65 | 0.55 |
| *Gulo gulo* | UAMZ 2622 | 0.63 | 0.48 |
| *Gulo gulo* | UAMZ 2625 | 0.64 | 0.47 |
| *Gulo gulo* | UAMZ 2628 | 0.64 | 0.47 |
| *Gulo gulo* | UAMZ 3480 | 0.65 | 0.47 |
| *Lynx canadensis* | UAMZ 3378 | 0.72 | 0.62 |
| *Lynx canadensis* | UAMZ 4400 | 0.72 | 0.66 |
| (Table S3 continued) |  |  |  |
| *Lynx canadensis* | UAMZ 5654 | 0.71 | 0.61 |
| *Lynx canadensis* | UAMZ 6695 | 0.73 | 0.68 |
| *Lynx canadensis* | UAMZ 6890 | 0.73 | 0.64 |
| *Lynx rufus* | UAMZ 10442 | 0.73 | 0.60 |
| *Lynx rufus* | UAMZ 2669 | 0.67 | 0.60 |
| *Lynx rufus* | UAMZ 3410 | 0.75 | 0.64 |
| *Lynx rufus* | UAMZ 9033 | 0.70 | 0.61 |
| *Martes pennanti* | UAMZ 1197 | 0.52 | 0.50 |
| *Martes pennanti* | UAMZ 2570 | 0.53 | 0.50 |
| *Martes pennanti* | UAMZ 2573 | 0.64 | 0.56 |
| *Martes pennanti* | UAMZ 2574 | 0.53 | 0.46 |
| *Martes pennanti* | UAMZ 3166 | 0.60 | 0.46 |
| *Martes pennanti* | UAMZ 3243 | 0.55 | 0.48 |
| *Martes pennanti* | UAMZ 9205 | 0.60 | 0.46 |
| *Martes pennanti* | UAMZ 9295 | 0.60 | 0.55 |
| *Martes pennanti* | UAMZ 9318 | 0.58 | 0.46 |
| *Mephitis mephitis* | UAMZ 2631 | 0.63 | 0.57 |
| *Mephitis mephitis* | UAMZ 3188 | 0.64 | 0.58 |
| *Mephitis mephitis* | UAMZ 3200 | 0.65 | 0.52 |
| *Mephitis mephitis* | UAMZ 6886 | 0.63 | 0.51 |
| *Mustela frenata* | UAMZ 1128 | 0.60 | 0.39 |
| *Mustela frenata* | UAMZ 3737 | 0.57 | 0.48 |
| *Mustela frenata* | UAMZ 5351 | 0.58 | 0.45 |
| *Mustela frenata* | UAMZ 5707 | 0.61 | 0.46 |
| *Mustela frenata* | UAMZ 78 | 0.58 | 0.48 |
| *Mustela frenata* | UAMZ 9245 | 0.57 | 0.46 |
| *Mustela frenata* | UAMZ 989 | 0.55 | 0.40 |
| *Mustela nivalis* | UAMZ 3170 | 0.48 | 0.45 |
| *Neovison vison* | UAMZ 3694 | 0.62 | 0.46 |
| *Neovison vison* | UAMZ 7858 | 0.57 | 0.44 |
| *Procyon lotor* | UAMZ 5760 | 0.67 | 0.52 |
| *Procyon lotor* | UAMZ 9031 | 0.71 | 0.55 |
| *Procyon lotor* | UAMZ A59 | 0.66 | 0.51 |
| *Puma concolor* | UAMZ 2644 | 0.72 | 0.66 |
| *Puma concolor* | UAMZ 2648 | 0.69 | 0.56 |
| *Puma concolor* | UAMZ 2649 | 0.71 | 0.59 |
| *Puma concolor* | UAMZ 2652 | 0.74 | 0.62 |
| *Puma concolor* | UAMZ 2653 | 0.72 | 0.57 |
| *Puma concolor* | UAMZ 3203 | 0.71 | 0.54 |
| *Puma concolor* | UAMZ 3204 | 0.71 | 0.62 |
| (Table S3 continued) |  |  |  |
| *Puma concolor* | UAMZ 3205 | 0.71 | 0.56 |
| *Puma concolor* | UAMZ 4210 | 0.69 | 0.55 |
| *Taxidea taxus* | UAMZ 2629 | 0.66 | 0.49 |
| *Taxidea taxus* | UAMZ 3181 | 0.72 | 0.48 |
| *Taxidea taxus* | UAMZ 3272 | 0.69 | 0.50 |
| *Taxidea taxus* | UAMZ 5352 | 0.66 | 0.47 |
| *Taxidea taxus* | UAMZ 6817 | 0.69 | 0.46 |
| *Taxidea taxus* | UAMZ 9291 | 0.69 | 0.56 |
| *Urocyon cinereoargenteus* | UAMZ 597 | 0.60 | 0.49 |
| *Ursus americanus* | UAMZ 2506 | 0.63 | 0.41 |
| *Ursus americanus* | UAMZ 2507 | 0.55 | 0.45 |
| *Ursus americanus* | UAMZ 2509 | 0.60 | 0.40 |
| *Ursus americanus* | UAMZ 2513 | 0.58 | 0.43 |
| *Ursus americanus* | UAMZ 2518 | 0.55 | 0.38 |
| *Ursus americanus* | UAMZ 2519 | 0.58 | 0.40 |
| *Ursus americanus* | UAMZ 2522 | 0.62 | 0.42 |
| *Ursus americanus* | UAMZ 2528 | 0.57 | 0.44 |
| *Ursus americanus* | UAMZ 3287 | 0.56 | 0.43 |
| *Ursus americanus* | UAMZ 5374 | 0.61 | 0.42 |
| *Ursus arctos* | UAMZ 2523 | 0.54 | 0.39 |
| *Ursus arctos* | UAMZ 2524 | 0.52 | 0.39 |
| *Ursus arctos* | UAMZ 2525 | 0.62 | 0.44 |
| *Ursus arctos* | UAMZ 2535 | 0.61 | 0.42 |
| *Ursus arctos* | UAMZ 3613 | 0.53 | 0.38 |
| *Ursus arctos* | UAMZ 3698 | 0.56 | 0.41 |
| *Ursus arctos* | UAMZ 3700 | 0.53 | 0.40 |
| *Ursus arctos* | UAMZ 3701 | 0.67 | 0.45 |
| *Ursus arctos* | UAMZ 4026 | 0.57 | 0.38 |
| *Vulpes vulpes* | UAMZ 2485 | 0.54 | 0.44 |
| *Vulpes vulpes* | UAMZ 2486 | 0.55 | 0.43 |
| *Vulpes vulpes* | UAMZ 2487 | 0.52 | 0.48 |
| *Vulpes vulpes* | UAMZ 2491 | 0.53 | 0.45 |
| *Vulpes vulpes* | UAMZ 2492 | 0.50 | 0.46 |
| *Vulpes vulpes* | UAMZ 2493 | 0.51 | 0.47 |
| *Vulpes vulpes* | UAMZ 2494 | 0.56 | 0.47 |
| *Vulpes vulpes* | UAMZ 344 | 0.57 | 0.45 |
| *Vulpes vulpes* | UAMZ 6684 | 0.54 | 0.50 |
| *Vulpes vulpes* | UAMZ 9181 | 0.57 | 0.52 |
| *Vulpes vulpes* | UAMZ 9208 | 0.53 | 0.50 |
