# Supplementary material for: Testing Adaptive Hypotheses of Convergence with Functional Landscapes: A Case Study of Bone-Cracking Hypercarnivores
Source: PLoS One. 2013 May 29;8(5):e65305. doi: 10.1371/journal.pone.0065305 (PMC3667121; doi:10.1371/journal.pone.0065305)
Supplement: Table S4 — Cranium ratio measurements of extant east African carnivorans. For abbreviations see Table S1 legend. (DOC) [file pone.0065305.s004.doc]

**Table S4. Cranium ratio measurements of extant east African carnivorans.**

| Taxon | Specimen # | W:L | D:L |
| --- | --- | --- | --- |
| *Acinonyx jubatus* | LACM 30788 | 0.91 | 0.65 |
| *Acinonyx jubatus* | LACM 51559 | 0.79 | 0.67 |
| *Atilax paludinosus* | LACM 45746 | 0.56 | 0.45 |
| *Atilax paludinosus* | LACM 53750 | 0.55 | 0.46 |
| *Atilax paludinosus* | LACM 53751 | 0.54 | 0.46 |
| *Atilax paludinosus* | LACM 53753 | 0.54 | 0.46 |
| *Atilax paludinosus* | LACM 53754 | 0.51 | 0.49 |
| *Bdeogale crassicauda* | LACM 42940 | 0.57 | 0.50 |
| *Bdeogale crassicauda* | LACM 42941 | 0.55 | 0.52 |
| *Bdeogale crassicauda* | LACM 42942 | 0.53 | 0.46 |
| *Bdeogale crassicauda* | LACM 56749 | 0.50 | 0.47 |
| *Bdeogale crassicauda* | LACM 56750 | 0.53 | 0.44 |
| *Canis aureus* | LACM 14479 | 0.56 | 0.46 |
| *Canis aureus* | LACM 52230 | 0.55 | 0.43 |
| *Canis aureus* | LACM 56747 | 0.51 | 0.45 |
| *Canis aureus* | LACM 56748 | 0.54 | 0.52 |
| *Canis aureus* | LACM 70167 | 0.53 | 0.46 |
| *Canis aureus* | LACM 70168 | 0.52 | 0.50 |
| *Canis aureus* | LACM 70169 | 0.51 | 0.47 |
| *Canis aureus* | LACM 70170 | 0.51 | 0.46 |
| *Caracal caracal* | LACM 42383 | 0.69 | 0.50 |
| *Civettictis civetta* | LACM 42943 | 0.48 | 0.43 |
| *Civettictis civetta* | LACM 53733 | 0.52 | 0.57 |
| *Civettictis civetta* | LACM 53734 | 0.50 | 0.40 |
| *Civettictis civetta* | LACM 71360 | 0.50 | 0.43 |
| *Crocuta crocuta* | LACM 30655 | 0.65 | 0.52 |
| *Crocuta crocuta* | MVZ 124188 | 0.61 | 0.49 |
| *Crocuta crocuta* | MVZ 124259 | 0.64 | 0.51 |
| *Crocuta crocuta* | MVZ 165159 | 0.67 | 0.55 |
| *Crocuta crocuta* | MVZ 165160 | 0.66 | 0.50 |
| *Crocuta crocuta* | MVZ 165161 | 0.67 | 0.53 |
| *Crocuta crocuta* | MVZ 165162 | 0.64 | 0.53 |
| *Crocuta crocuta* | MVZ 165163 | 0.62 | 0.57 |
| *Crocuta crocuta* | MVZ 165165 | 0.69 | 0.58 |
| *Crocuta crocuta* | MVZ 165167 | 0.63 | 0.58 |
| *Crocuta crocuta* | MVZ 165168 | 0.64 | 0.53 |
| *Crocuta crocuta* | MVZ 165169 | 0.62 | 0.59 |
| *Crocuta crocuta* | MVZ 165170 | 0.63 | 0.53 |
| (Table S4 continued) |  |  |  |
| *Crocuta crocuta* | MVZ 165171 | 0.61 | 0.57 |
| *Crocuta crocuta* | MVZ 165172 | 0.64 | 0.55 |
| *Crocuta crocuta* | MVZ 165173 | 0.66 | 0.57 |
| *Crocuta crocuta* | MVZ 165174 | 0.64 | 0.53 |
| *Crocuta crocuta* | MVZ 165175 | 0.66 | 0.55 |
| *Crocuta crocuta* | MVZ 165176 | 0.67 | 0.56 |
| *Crocuta crocuta* | MVZ 165177 | 0.61 | 0.56 |
| *Crocuta crocuta* | MVZ 165179 | 0.64 | 0.59 |
| *Crocuta crocuta* | MVZ 165180 | 0.61 | 0.55 |
| *Crocuta crocuta* | MVZ 165181 | 0.65 | 0.57 |
| *Crocuta crocuta* | MVZ 165182 | 0.66 | 0.54 |
| *Crocuta crocuta* | MVZ 173733 | 0.64 | 0.62 |
| *Crocuta crocuta* | MVZ 173734 | 0.65 | 0.55 |
| *Crocuta crocuta* | MVZ 173736 | 0.60 | 0.47 |
| *Crocuta crocuta* | MVZ 173737 | 0.61 | 0.55 |
| *Crocuta crocuta* | MVZ 173738 | 0.62 | 0.55 |
| *Crocuta crocuta* | MVZ 173739 | 0.63 | 0.46 |
| *Crocuta crocuta* | MVZ 173740 | 0.62 | 0.52 |
| *Crocuta crocuta* | MVZ 173743 | 0.63 | 0.57 |
| *Crocuta crocuta* | MVZ 173751 | 0.62 | 0.58 |
| *Crocuta crocuta* | MVZ 173759 | 0.62 | 0.60 |
| *Crocuta crocuta* | MVZ 173762 | 0.70 | 0.51 |
| *Crocuta crocuta* | MVZ 173763 | 0.61 | 0.51 |
| *Crocuta crocuta* | MVZ 173764 | 0.62 | 0.59 |
| *Crocuta crocuta* | MVZ 173768 | 0.66 | 0.56 |
| *Crocuta crocuta* | MVZ 173771 | 0.66 | 0.61 |
| *Crocuta crocuta* | MVZ 173772 | 0.62 | 0.51 |
| *Crocuta crocuta* | MVZ 173773 | 0.65 | 0.55 |
| *Crocuta crocuta* | MVZ 175801 | 0.64 | 0.55 |
| *Crocuta crocuta* | MVZ 184088 | 0.61 | 0.57 |
| *Crocuta crocuta* | MVZ 184089 | 0.63 | 0.54 |
| *Crocuta crocuta* | MVZ 4823 | 0.65 | 0.52 |
| *Felis sylvestris* | LACM 14478 | 0.71 | 0.57 |
| *Felis sylvestris* | LACM 14480 | 0.76 | 0.58 |
| *Felis sylvestris* | LACM 41785 | 0.73 | 0.55 |
| *Felis sylvestris* | LACM 41786 | 0.70 | 0.58 |
| *Felis sylvestris* | LACM 41787 | 0.69 | 0.63 |
| *Felis sylvestris* | LACM 41788 | 0.72 | 0.59 |
| *Felis sylvestris* | LACM 45759 | 0.79 | 0.60 |
| *Genetta erlangeri* | UAMZ 4441 | 0.53 | 0.53 |
| (Table S4 continued) |  |  |  |
| *Genetta maculata* | LACM 36692 | 0.47 | 0.46 |
| *Genetta maculata* | LACM 36695 | 0.47 | 0.44 |
| *Genetta maculata* | LACM 42935 | 0.51 | 0.45 |
| *Genetta maculata* | LACM 42936 | 0.54 | 0.47 |
| *Genetta maculata* | LACM 42937 | 0.48 | 0.47 |
| *Genetta maculata* | LACM 42938 | 0.51 | 0.47 |
| *Genetta maculata* | LACM 42939 | 0.53 | 0.45 |
| *Genetta pardina* | UAMZ 3232 | 0.48 | 0.47 |
| *Genetta rubiginosa* | LACM 36693 | 0.52 | 0.46 |
| *Genetta rubiginosa* | LACM 36694 | 0.51 | 0.43 |
| *Genetta rubiginosa* | LACM 40153 | 0.54 | 0.51 |
| *Genetta rubiginosa* | LACM 40154 | 0.52 | 0.50 |
| *Genetta rubiginosa* | LACM 45750 | 0.50 | 0.49 |
| *Herpestes sanguineus* | LACM 36687 | 0.50 | 0.41 |
| *Herpestes sanguineus* | LACM 40134 | 0.52 | 0.49 |
| *Herpestes sanguineus* | LACM 45752 | 0.50 | 0.45 |
| *Herpestes sanguineus* | LACM 45753 | 0.50 | 0.46 |
| *Herpestes sanguineus* | LACM 53748 | 0.48 | 0.46 |
| *Herpestes sanguineus* | LACM 53749 | 0.49 | 0.48 |
| *Herpestes sanguineus* | LACM 56752 | 0.50 | 0.46 |
| *Herpestes sanguineus* | LACM 56753 | 0.50 | 0.45 |
| *Hyaena hyaena* | LACM 31264 | 0.70 | 0.58 |
| *Ichneumia albicauda* | LACM 40136 | 0.50 | 0.44 |
| *Ichneumia albicauda* | LACM 40137 | 0.49 | 0.45 |
| *Ictonyx* | LACM 42376 | 0.64 | 0.49 |
| *Ictonyx* | LACM 56703 | 0.58 | 0.46 |
| *Ictonyx* | LACM 56705 | 0.66 | 0.49 |
| *Lycaon pictus* | LACM 30587 | 0.64 | 0.58 |
| *Lycaon pictus* | LACM 30588 | 0.61 | 0.55 |
| *Lycaon pictus* | LACM 51145 | 0.63 | 0.52 |
| *Lycaon pictus* | LACM 72188 | 0.61 | 0.54 |
| *Lycaon pictus* | MVZ 117806 | 0.62 | 0.49 |
| *Lycaon pictus* | MVZ 124258 | 0.61 | 0.47 |
| *Lycaon pictus* | MVZ 184049 | 0.64 | 0.48 |
| *Lycaon pictus* | MVZ 4842 | 0.64 | 0.52 |
| *Mellivora capensis* | LACM 30135 | 0.64 | 0.48 |
| *Nandinia binotata* | LACM 53741 | 0.52 | 0.43 |
| *Nandinia binotata* | LACM 53742 | 0.51 | 0.48 |
| *Nandinia binotata* | LACM 53743 | 0.52 | 0.44 |
| *Nandinia binotata* | LACM 53744 | 0.60 | 0.46 |
| (Table S4 continued) |  |  |  |
| *Nandinia binotata* | LACM 53745 | 0.58 | 0.47 |
| *Otocyon megalotis* | LACM 41790 | 0.57 | 0.45 |
| *Otocyon megalotis* | LACM 41792 | 0.57 | 0.41 |
| *Otocyon megalotis* | LACM 41793 | 0.55 | 0.47 |
| *Otocyon megalotis* | LACM 59645 | 0.53 | 0.40 |
| *Otocyon megalotis* | LACM 60618 | 0.50 | 0.48 |
| *Otocyon megalotis* | LACM 62843 | 0.57 | 0.40 |
| *Otocyon megalotis* | LACM 62844 | 0.60 | 0.46 |
| *Panthera leo* | LACM 30786 | 0.63 | 0.60 |
| *Panthera leo* | LACM 31182 | 0.68 | 0.54 |
| *Panthera leo* | LACM 51294 | 0.67 | 0.53 |
| *Panthera leo* | LACM 51297 | 0.65 | 0.63 |
| *Panthera leo* | LACM 51553 | 0.60 | 0.57 |
| *Panthera leo* | LACM 51567 | 0.67 | 0.57 |
| *Panthera leo* | LACM 54393 | 0.66 | 0.57 |
| *Panthera leo* | LACM 30787 | 0.63 | 0.53 |
| *Panthera leo* | LACM 31068 | 0.63 | 0.55 |
| *Panthera leo* | LACM M567 | 0.68 | 0.50 |
| *Panthera leo* | MVZ 117848 | 0.66 | 0.52 |
| *Panthera leo* | MVZ 117849 | 0.66 | 0.61 |
| *Panthera leo* | MVZ 117850 | 0.64 | 0.59 |
| *Panthera leo* | MVZ 117851 | 0.67 | 0.59 |
| *Panthera leo* | MVZ 117852 | 0.64 | 0.55 |
| *Panthera leo* | MVZ 117853 | 0.60 | 0.57 |
| *Panthera leo* | MVZ 117854 | 0.65 | 0.64 |
| *Panthera leo* | MVZ 117856 | 0.65 | 0.66 |
| *Panthera leo* | MVZ 117858 | 0.63 | 0.57 |
| *Panthera leo* | MVZ 117859 | 0.62 | 0.67 |
| *Panthera leo* | MVZ 124260 | 0.66 | 0.63 |
| *Panthera leo* | MVZ 124261 | 0.72 | 0.64 |
| *Panthera leo* | MVZ 152836 | 0.65 | 0.59 |
| *Panthera leo* | MVZ 96804 | 0.66 | 0.63 |
| *Panthera pardus* | LACM 438 | 0.70 | 0.67 |
| *Panthera pardus* | LACM 51283 | 0.62 | 0.54 |
| *Panthera pardus* | LACM 51560 | 0.65 | 0.53 |
| *Panthera pardus* | LACM 54508 | 0.66 | 0.58 |
| *Panthera pardus* | LACM 648 | 0.65 | 0.61 |
| *Panthera pardus* | MVZ 117843 | 0.66 | 0.51 |
| *Panthera pardus* | MVZ 126802 | 0.66 | 0.54 |
| *Proteles cristatus* | LACM 60619 | 0.62 | 0.44 |
| (Table S4 continued) |  |  |  |
| *Proteles cristatus* | MVZ 117841 | 0.65 | 0.43 |
| *Proteles cristatus* | UAMZ 10470 | 0.57 | 0.44 |
