# Supplementary material for: Testing Adaptive Hypotheses of Convergence with Functional Landscapes: A Case Study of Bone-Cracking Hypercarnivores
Source: PLoS One. 2013 May 29;8(5):e65305. doi: 10.1371/journal.pone.0065305 (PMC3667121; doi:10.1371/journal.pone.0065305)
Supplement: Table S5 — Theoretical models and their parameters. D∶L, skull depth to length ratio; W∶L, skull width to length ratio; elements: number of four-noded tetrahedral finite elements in model; SE, skull strain energy (in Joules); adjSE, strain energy adjusted by model volume; Fout, output bite force (in Newtons); MA, mechanical advantage; S.T., solution time required for FEA (in minutes). Model files are deposited in Dryad (doi:10.5061/dryad.r2b1h). (DOC) [file pone.0065305.s005.doc]

**Table S5. Theoretical models and their parameters.** D:L, skull depth to length ratio; W:L, skull width to length ratio; elements: number of four-noded tetrahedral finite elements in model; SE, skull strain energy (in Joules); adjSE, strain energy adjusted by model volume; Fout, output bite force (in Newtons); MA, mechanical advantage; S.T., solution time required for FEA (in minutes). Model files are deposited in Dryad (doi:10.5061/dryad.r2b1h).

| Model name | D:L | W:L | Elements | SE (J) | adjSE (J) | Fout (N) | MA | S.T. (min) |
| --- | --- | --- | --- | --- | --- | --- | --- | --- |
| J022611T38 | 0.33 | 0.42 | 1,139,265 | 3.39 | 3.30 | 7653.53 | 0.19 | 63 |
| J022611T39 | 0.33 | 0.55 | 1,177,734 | 3.10 | 3.05 | 7347.61 | 0.18 | 90 |
| J022611T40 | 0.33 | 0.69 | 1,138,486 | 2.67 | 2.63 | 6999.04 | 0.18 | 73 |
| J022611T41 | 0.33 | 0.83 | 1,086,419 | 3.27 | 3.21 | 6737.50 | 0.17 | 78 |
| J022611T42 | 0.33 | 0.97 | 1,047,996 | 3.02 | 2.93 | 6442.83 | 0.16 | 65 |
| J022611T43 | 0.33 | 1.11 | 1,007,072 | 2.83 | 2.72 | 5932.73 | 0.15 | 43 |
| J022611T33 | 0.38 | 0.42 | 1,146,486 | 4.20 | 4.10 | 8960.00 | 0.23 | 108 |
| J021411T01 | 0.38 | 0.55 | 1,203,707 | 3.75 | 3.75 | 8635.51 | 0.22 | 97 |
| J030211T48 | 0.38 | 0.69 | 1,153,383 | 3.67 | 3.70 | 8313.37 | 0.21 | 70 |
| J021511T03 | 0.38 | 0.83 | 1,103,659 | 3.61 | 3.64 | 7966.53 | 0.20 | 57 |
| J021711T08 | 0.38 | 0.97 | 1,072,618 | 3.01 | 3.02 | 7768.39 | 0.20 | 64 |
| J021711T09 | 0.38 | 1.11 | 1,044,292 | 3.33 | 3.32 | 7401.19 | 0.19 | 44 |
| J022611T34 | 0.51 | 0.42 | 1,095,971 | 5.81 | 5.62 | 9436.23 | 0.24 | 77 |
| J021711T10 | 0.51 | 0.55 | 1,124,466 | 5.31 | 5.32 | 9919.35 | 0.25 | 69 |
| J021811T14 | 0.51 | 0.69 | 1,108,403 | 4.16 | 4.22 | 9768.02 | 0.25 | 66 |
| J021811T15 | 0.51 | 0.83 | 1,095,138 | 3.60 | 3.67 | 9275.19 | 0.23 | 72 |
| J021811T16 | 0.51 | 0.97 | 1,094,398 | 3.51 | 3.57 | 9116.86 | 0.23 | 72 |
| J021811T17 | 0.51 | 1.11 | 1,069,458 | 2.99 | 3.03 | 8496.61 | 0.21 | 47 |
| J022611T35 | 0.59 | 0.42 | 1,030,327 | 6.17 | 5.91 | 10263.06 | 0.26 | 67 |
| J021711T11 | 0.59 | 0.55 | 1,077,924 | 5.96 | 5.94 | 10127.58 | 0.25 | 67 |
| J030211T46 | 0.59 | 0.69 | 1,081,675 | 5.05 | 5.13 | 10029.38 | 0.25 | 70 |
| J022011T21 | 0.59 | 0.83 | 1,103,739 | 5.82 | 5.95 | 9428.57 | 0.24 | 74 |
| J030211T47 | 0.59 | 0.97 | 1,129,459 | 3.97 | 4.06 | 9717.49 | 0.24 | 68 |
| J021911T18 | 0.59 | 1.11 | 1,096,699 | 4.54 | 4.64 | 9269.83 | 0.23 | 75 |
| J030311T49 | 0.65 | 0.42 | 995,611 | 6.00 | 5.68 | 10898.32 | 0.27 | 56 |
| J021811T12 | 0.65 | 0.55 | 1,049,741 | 6.62 | 6.53 | 11338.75 | 0.28 | 48 |
| J022111T26 | 0.65 | 0.69 | 1,085,106 | 4.71 | 4.74 | 10571.10 | 0.27 | 63 |
| J022111T25 | 0.65 | 0.83 | 1,127,189 | 5.74 | 5.85 | 10200.98 | 0.26 | 81 |
| J022011T23 | 0.65 | 0.97 | 1,182,948 | 4.96 | 5.07 | 9366.17 | 0.24 | 86 |
| J022011T19 | 0.65 | 1.11 | 1,134,838 | 5.04 | 5.16 | 10320.85 | 0.26 | 74 |
| J030211T45 | 0.73 | 0.42 | 962,202 | 7.09 | 6.63 | 10939.08 | 0.27 | 45 |
| J021811T13 | 0.73 | 0.55 | 1,032,173 | 7.93 | 7.76 | 11725.13 | 0.29 | 49 |
| J022111T28 | 0.73 | 0.69 | 1,092,655 | 6.09 | 6.10 | 11245.83 | 0.28 | 62 |
| J022111T27 | 0.73 | 0.83 | 1,167,406 | 5.54 | 5.63 | 10570.20 | 0.27 | 80 |
| (Table S5 continued) |  |  |  |  |  |  |  |  |
| J030211T44 | 0.73 | 0.97 | 1,248,480 | 5.45 | 5.56 | 10679.10 | 0.27 | 100 |
| J021611T06 | 0.73 | 1.11 | 1,186,504 | 5.61 | 5.74 | 10949.00 | 0.27 | 90 |
