# Supplementary material for: Testing Adaptive Hypotheses of Convergence with Functional Landscapes: A Case Study of Bone-Cracking Hypercarnivores
Source: PLoS One. 2013 May 29;8(5):e65305. doi: 10.1371/journal.pone.0065305 (PMC3667121; doi:10.1371/journal.pone.0065305)
Supplement: Table S6 — Finite element model parameters of actual fossil and extant carnivorans analyzed in the study. The number of elements were kept as close to 1,000,000 elements as possible for both actual and theoretical models. Three models had lower counts of elements (P. brunnea, P. cristata, C. lupus), but no correlation of MA or SE values to lower element counts was detected. Abbreviations as in Table S5. Model files are deposited in Dryad (doi:10.5061/dryad.r2b1h). (DOC) [file pone.0065305.s006.doc]

**Table S6. Finite element model parameters of actual fossil and extant carnivorans analyzed in the study.** The number of elements were kept as close to 1,000,000 elements as possible for both actual and theoretical models. Three models had lower counts of elements (*P. brunnea, P. cristata, C. lupus*), but no correlation of MA or SE values to lower element counts was detected. Abbreviations as in Table S5. Model files are deposited in Dryad (doi:10.5061/dryad.r2b1h).

| Model name | D:L | W:L | Elements | SE (J) | adjSE (J) | Fout (N) | MA |
| --- | --- | --- | --- | --- | --- | --- | --- |
| *Crocuta crocuta* | 0.44 | 0.66 | 1,173,850 | 4.49 | 3.87 | 10743.08 | 0.27 |
| *Parahyaena brunnea* | 0.43 | 0.65 | 537,232 | 4.35 | 3.82 | 10601.93 | 0.27 |
| *Ikelohyaena abronia* | 0.38 | 0.63 | 1,188,737 | 5.30 | 4.50 | 8503.25 | 0.21 |
| *Chasmaporthetes lunensis* | 0.48 | 0.66 | 1,121,102 | 4.94 | 4.93 | 9387.11 | 0.24 |
| *Ictitherium sp.* | 0.38 | 0.55 | 1,203,707 | 3.75 | 3.75 | 8635.51 | 0.22 |
| *Proteles cristata* | 0.34 | 0.65 | 453,670 | 3.88 | 3.11 | 4834.74 | 0.12 |
| *Borophagus secundus* | 0.53 | 0.71 | 920,143 | 3.49 | 3.16 | 9280.70 | 0.23 |
| *Epicyon haydeni* | 0.44 | 0.71 | 937,061 | 2.75 | 2.77 | 7706.61 | 0.19 |
| *Microtomarctus conferta* | 0.42 | 0.66 | 948,735 | 3.07 | 2.79 | 7508.95 | 0.19 |
| *Canis lupus* | 0.38 | 0.58 | 679,111 | 5.79 | 4.46 | 9121.44 | 0.23 |
| *Lycaon pictus* | 0.41 | 0.67 | 1,134,781 | 4.24 | 3.72 | 6668.69 | 0.17 |
| *Mesocyon coryphaeus* | 0.35 | 0.62 | 1,115,623 | 4.35 | 3.92 | 8982.51 | 0.23 |
| *Dinocrocuta gigantea* | 0.44 | 0.74 | 1,532,146 | 6.38 | 6.66 | 10551.26 | 0.26 |
